# Integrated Bioinformatic Analysis of DNA Methylation and Immune Infiltration in Endometrial Cancer

**DOI:** 10.1155/2022/5119411

**Published:** 2022-06-20

**Authors:** Fangfang Dai, Jinglin Wu, Zhimin Deng, Hengxing Li, Wei Tan, Mengqin Yuan, Dongyong Yang, Shiyi Liu, Yajing Zheng, Min Hu, Chaoyan Yuan, Yanxiang Cheng

**Affiliations:** ^1^Department of Obstetrics and Gynecology, Renmin Hospital of Wuhan University, Wuhan, Hubei 430060, China; ^2^Department of Gynecology, Minda Hospital of Hubei Minzu University, Enshi, China

## Abstract

**Background:**

Endometrial cancer greatly threatens the health of female. Emerging evidences have demonstrated that DNA methylation and immune infiltration are involved in the occurrence and development of endometrial cancer. However, the mechanism and prognostic biomarkers of endometrial cancer are still unclear. In this study, we assess DNA methylation and immune infiltration via bioinformatic analysis.

**Methods:**

The latest RNA-Seq, DNA methylation data, and clinical data related to endometrial cancer were downloaded from the UCSC Xena dataset. The methylation-driven genes were selected, and then the risk score was obtained using “MethylMix” and “corrplot” R packages. The connection between methylated genes and the expression of screened driven genes were explored using “survminer” and “beeswarm” packages, respectively. Finally, the role of *VTCN1*in immune infiltration was analyzed using “CIBERSORT” package.

**Results:**

In this study, 179 upregulated genes, and 311 downregulated genes were identified and found to be related to extracellular matrix organization, cell–cell junctions, and cell adhesion molecular binding. The methylation-driven gene *VTCN1* was selected, and patients classified to the hypomethylation and high expression group displayed poor prognosis. The *VTCN1* gene exhibited highest correlation coefficient between methylation and expression. More importantly, the hypomethylation of promoter of *VTCN1* led to its high expression, thereby induce tumor development by inhibiting CD8+ T cell infiltration.

**Conclusions:**

Overall, our study was the first to reveal the mechanism of endometrial cancer by assessing DNA methylation and immune infiltration via integrated bioinformatic analysis. In addition, we found a pivotal prognostic biomarker for the disease. Our study provides potential targets for the diagnosis and prognosis of endometrial cancer in the future.

## 1. Introduction

Endometrial cancer, the second most common female malignancy, greatly threatens the health of women. According to the statistics in 2018, the numbers of new cases and deaths in the world were over 380,000 and 89,000, respectively [1]. With the increase in the prevalence of obesity and ageing population, the incidence of endometrial cancer is increasing [2]. Approximately 70% of endometrial cancer are confined to uterus when diagnosed; these cases are mainly treated by removing the uterus, which is associated with better prognosis and approximately 75% survival rate[3, 4]. However, 10~15% of endometrial cancer extend beyond the uterine tissues when diagnosed. The survival rate in these cases is less than 5 ~15% [5]. It is highly desirable to develop novel targets for the intervention and treatment of endometrial cancer patients.

Cancer is generally considered to be an epigenetic disease caused by abnormal gene expression. The epigenetic alternation plays a critical role in the progression of endometrial cancer [6, 7]. DNA methylation, a common kind of epigenetic change, can activate or silence some genes to promote or inhibit related signaling pathways [8]. Abnormal DNA methylation, including hyper- and hypomethylation, can both result in the occurrence of disease [9]. DNA methylation in cancers, such as lung cancer [10], prostate cancer [11], and breast cancer [12], have been widely studied.

With the development of single-cell technology, the role of tumor microenvironment (TME), which is consisted of immune cells, fibroblasts, endothelial cells, inflammatory mediators, and extracellular matrix, has attracted increasing attention in the study of endometrial cancer [13, 14]. Immunoresponse is an important factor for the prognostic value in endometrial cancer [15]. Usually, the increased number of cytotoxic T lymphocytes (CTLs, CD8^+^ T cells) is an independent prognostic indicator for better prognosis in endometrial cancer [15]. CIBERSORT is broadly performed to explore the abundance of immune cells in normal and tumor tissues [16, 17]. Many recent studies have examined the role of immune cells in TME in the mechanism of cancers, such as prostate cancer [18], clear cell renal cell carcinoma [19], and endometrial cancer [20]. However, few have studied the disease mechanism and prognosis biomarkers related to DNA methylation and immune cell infiltration.

In this study, we explored the prognostic biomarkers of DNA methylation and immune cell infiltration. *VTCN1*, exhibits est correlation coefficient between methylation and expression, was identified. *VTCN1* (a T cell activation suppressor 1), also known as B7-H4, can regulate T cell activation in non-small-cell lung cancer [21], hepatocellular carcinoma [22], and prostate cancer [23]. To further explore whether *VTCN1* is involved in regulating the occurrence and development of endometrial cancer through immune factors, we analyzed the level of immune cell infiltration in normal and tumor tissues. The results showed that *VTCN1* is indeed negatively correlated with CD8^+^ T cells in endometrial cancer, and there was lower T cell infiltration in tumor tissues in the high *VTCN1* expression group. Finally, we demonstrated that *VTCN1* was upregulated in tumors, and hypomethylation and high expression of *VTCN1* were associated with poor prognosis. Our study demonstrated that *VTCN1* may be involved in the occurrence and development of endometrial cancer by inhibiting CD8^+^ T cell infiltration. This finding may contribute to understand the mechanism of disease and provide a prognostic biomarker for endometrial cancer.

## 2. Methods

### 2.1. Data Download and Preprocessing

The latest RNA-Seq (35 normal samples and 422 tumor samples) and DNA methylation data (46 normal samples and 422 tumor samples) were downloaded from the UCSC Xena dataset (https://xenabrowser.net/datapages/). The data were processed and normalized via *R* software. The differentially expressed genes (DEGs) were analyzed using the “limma” package. The DEGs were screened out with the criteria of |logFC| > 2 and adj-*P* value < 0.05. Gene Ontology (GO) function and Kyoto Encyclopedia of Genes and Genomes (KEGG) pathways were analyzed using the “org.Hs.e.g.db” package. A value of *P* < 0.05 was defined as statistically significant.

### 2.2. Screening for Driven Genes

Generally, the “MethylMix” package was used to explore the driven methylated genes. For transcriptomic profiling, differential expression analysis was performed on TCGA RNA-seq data that matched mDNA profiles. Because methylated genes usually negatively regulate their mRNA, it showed 13 driven differentially methylated genes (DEMs), including 2 downregulated (*RP11-469H8.6* and *VTCN1*) and 11 upregulated methylated genes (*KLF9*, *PGR*, *DDR2*, *TSPYL5*, *FAXDC2*, *HSPB6*, *GYPC*, *CDO1*, *C8orf88*, *TMEM132C*, and *WT1-AS*).

### 2.3. The Risk Score of the Above Genes

The clinical data were downloaded from the UCSC Xena dataset. Risk scores were calculated via the “survival” package. The survival curve and heat map were generated based on the high- and low-risk scores. Subsequently, 5 genes were selected, including *TSPYL5*, *KLF9*, *GYPC*, *VTCN1*, and *PGR*. The risk score for each patient was performed as our previous article [24]: risk score = *b* gene (1) × *E* gene (1) + *β* gene (2) × *E* gene (2) + ⋯+*β* gene (*n*) × *E* gene (*n*).

*E* denotes the normalized expression level of the gene, and *b* denotes the corresponding regression coefficient.

### 2.4. Survival Analysis

A survival analysis including the highly methylated genes and genes with low expression was implemented in *R* software.

### 2.5. The Evaluation of the Protein Expression

The expression of *GYPC, VTCN1*, and *PGR* genes in the paired tumor and normal groups was analyzed by the “limma” and “ggpubr” packages. The protein expression was analyzed by The Human Protein Atlas (https://www.proteinatlas.org/).

### 2.6. Immune Cell Infiltration

CIBERSORT is a deconvolution algorithm that uses a set of reference gene-expression values (a signature with 547 genes) considered a minimal representation for each cell type. Based on those values, cell type proportions in data from bulk tumor samples with mixed cell types are inferred using support vector regression. CIBERSORT can be applied to distinguish 22 human immune cells, including B cells, T cells, NK cells, macrophages, DCs, and myeloid subsets, based on the high specificity and sensitivity of the gene expression profile. To determine whether there is a correlation between tumor immune cells infiltrationand immune-related gene expression, tumor infiltration with six types of immune cells (CD4^+^ T cells, CD8^+^ T cells, B cells, neutrophils, macrophages, mast cells, and dendritic cells) was analyzed by CIBERSORT.

### 2.7. The *VTCN1* Gene Expression

The genes related to *VTCN1* were selected via the STRING database (https://string-db.org/). The GO pathways of these genes were analyzed using the Metascape dataset (http://metascape.org/gp/index.html#/main/step1). The expression of *VTCN1* was divided into two groups according to the wilcox test function. A visualization of the correction of*VTCN1*expression with immune cells was generated using the “vioplot” package. Six patients with advanced endometrial cancer and normal tissues were collected. Fresh tissues are stored at -80°C for quantitative analysis. As we recently published, [25] TRIzol (Invitrogen) 1 mL was added into 100 mg tissues, and RNA was further extracted by chloroform, isopropyl alcohol, and ethanol. Subsequently, reverse transcription and quantitative analysis were performed according to the protocol of kit (Shanghai Yisheng Co., Ltd.). Primers for PCR are as follows:

VTCN1_FGAATCGGAGATCAAAAGGC

VTCN1_RGCTGATGGCAAAGAAAGAA

PGR_1FCAAGCCCTAAGCCAGAGA

PGR_1RCAGCAAAGAACTGGAGGTG

GYPC_1FCGTGTGGAGCTTCCTGTCT

GYPC_1RAGGCTCTGCAATGGTGGT

GAPDH_FGGAGTCCACTGGCGTCTTCA

GAPDH_RGTCATGAGTCCTTCCACGATACC

### 2.8. Statistical Analysis and Visualization

The raw data were collated by Practical Extraction and Report Language (Perl, version 5.30.0) and *R* software (version 4.0.3). The statistical analysis and visualization of the statistical results were implemented with *R* software and Cytoscape (version 3.8.0).

## 3. Results

### 3.1. Functional Analysis of DEGs

**Figure 1:**
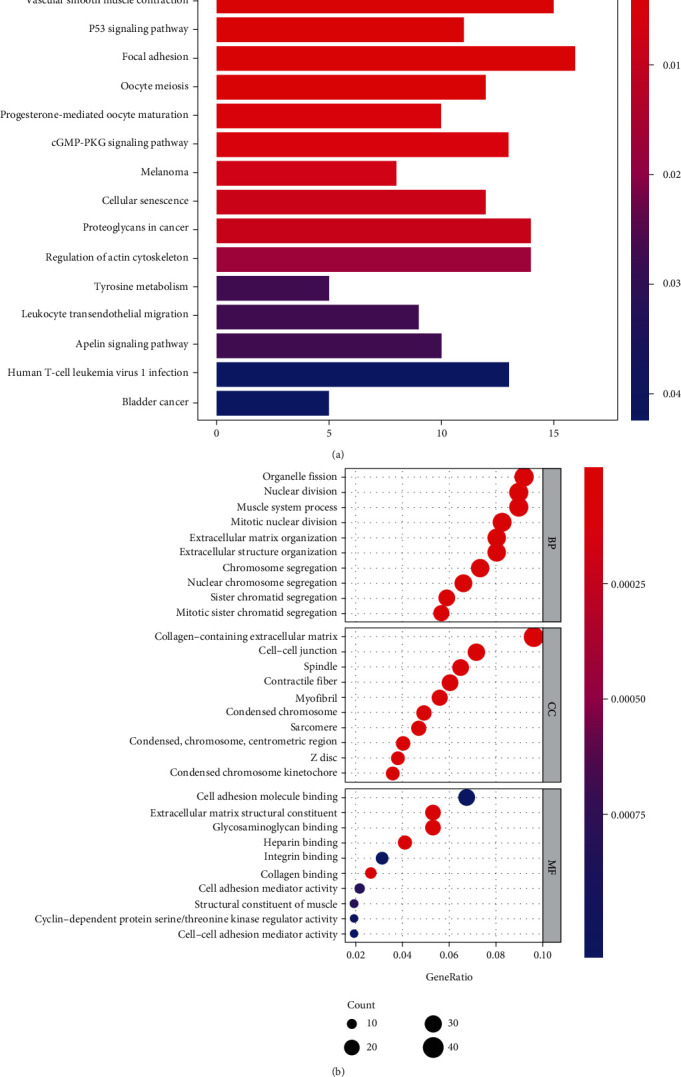
The map of (a) KEGG pathways and (b) GO function.

According to the criteria of |logFC| > 2 and adj-*P* value < 0.05, there were 490 DEGs, among which there were 179 upregulated genes and 311 downregulated genes. The volcano plot is shown in Figure S1. Second, Gene Ontology (GO) function and Kyoto Encyclopedia of Genes and Genomes (KEGG) pathway analyses were performed via *R* software. The results indicated that endometrial cancer was involved in the cell cycle, P53 signaling pathway, and focal adhesion in KEGG pathway **(**Figure 1(a)**)**. GO function included biological process (BP), cellular component (CC), and molecular function (MF). The GO analysis showed that endometrial cancer was related to extracellular matrix organization, cell–cell junctions, and cell adhesion molecular binding **(**Figure 1(b)**)**.

### 3.2. The Methylation-Driven Genes

**Figure 2:**
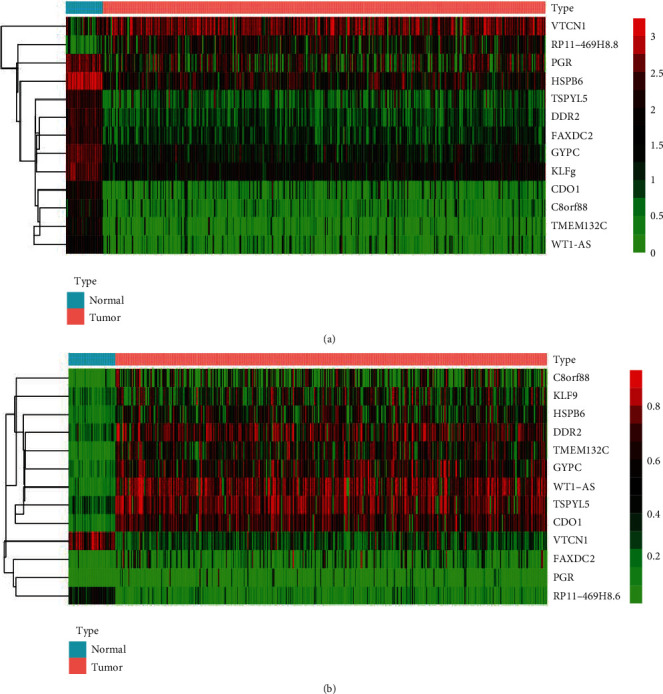
(a) The expressed heat map and (b) methylated heat map of methylated driven genes.

It is well documented that there is inverse correlation between DNA methylation and mRNA levels. In the next step, the methylation-driven genes were selected using the “MethylMix” and “Corrplot” packages in *R* software. There were 13 methylation-driven genes: *WT1-AS*, *CDO1*, *RP11-469H8.6*, *TMEM132C*, *GYPC*, *TSPYL5*, *VTCN1*, *DDR2*, *HSPB6*, *KLF9*, *C8orf88*, *FAXDC2*, and *PGR.* Among them, the expression of *RP11-469H8.6* and *VTCN1* was upregulated in tumors, and the others were downregulated. The mRNA expressed and DNA methylated heat map of above 13 DNA are displayed in Figures 2(a) and 2(b) (|*R*| > 0.3, *P* < 0.05).

### 3.3. Survival Analysis

**Figure 3:**
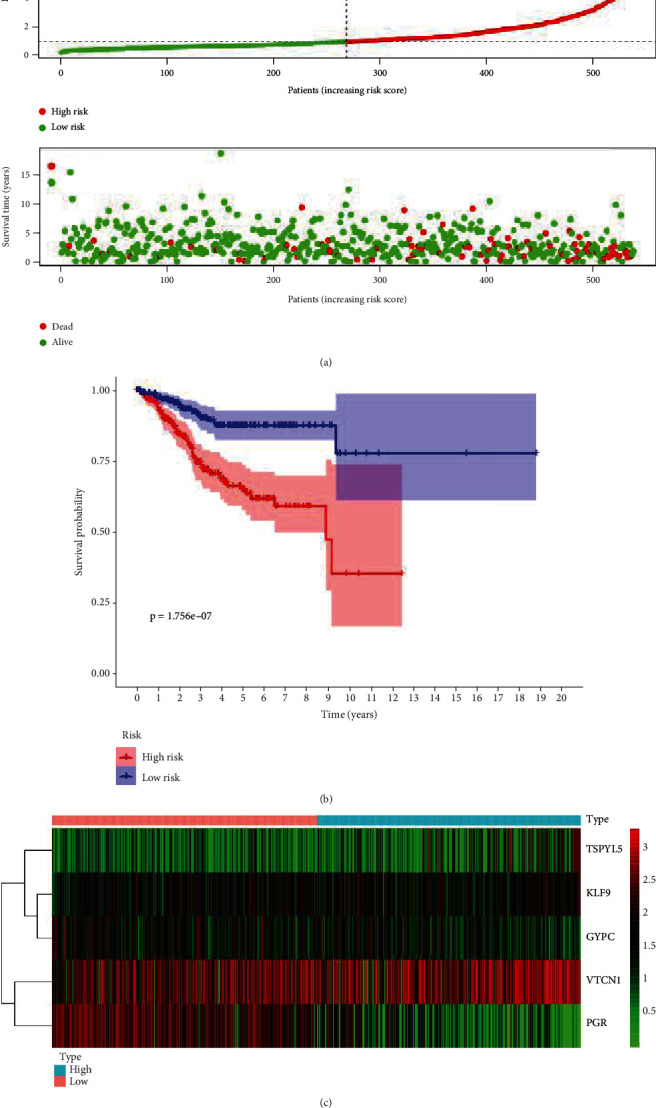
(a) The risk score of survival time. (b) The survival analysis of high-risk score and low risk. (c) The expressed heat map of *TSPYL5*, *KLF9*, *GYPC*, *VTCN1*, and *PGR* according to the risk score.

**Figure 4:**
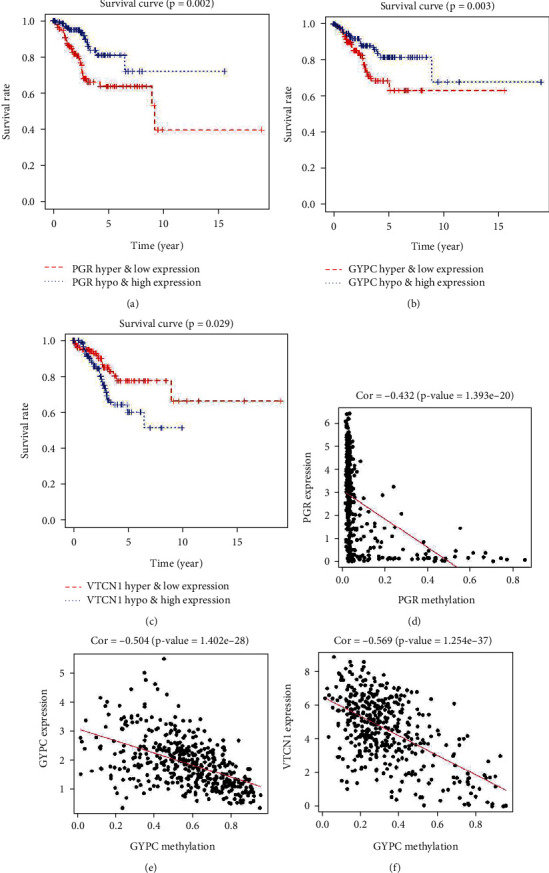
The survival curve of gene hyper methylation and low expression or hypomethylation and high expression (a) *PGR*, (b) *GYPC*, and (c) *VTCN1*. The correction of gene expression and methylation (d) *PGR*, (e) *GYPC*, and (f) *VTCN1*.

Survival analysis was performed with the product-limit method (Kaplan–Meier analysis). Additionally, the log-rank test (Mantel-Cox test) was used to compare the difference in the survival status between the high- (268 patients) and low-risk groups (269 patients) using the “Survminer” package. The Cox model was applied to build a risk model to obtain the risk value of patients, and the following genes were identified: *TSPYL5*, *KLF9*, *GYPC*, *VTCN1*, and *PGR*. The classification of patients was based on the median value of the risk score **(**Figure 3(a)**)**. Obviously, a lower risk indicates a better prognosis, and a higher risk indicates a poorer prognosis (Figure 3(b)). The heat map of the *TSPYL5*, *KLF9*, *GYPC*, *VTCN1*, and *PGR* expression is shown in Figure 3(c). Patients were classified to a high expression group and a low expression group based on the expression levels of *PGR*, *GYPC*, and *VTCN1*. The survival curves of the above 13 methylation-driven genes were generated using survival software. Three genes (*PGR, VTCN1*, and *GYPC*) were considered statistically significant (*P* < 0.05) (Figures 4(a)–4(c)). The hypomethylation and high expression group of *VTCN1* displays poor prognosis, while the lower expression group showed better prognosis (*P* < 0.05). The negative correlations of *PGR*, *VTCN1*, and *GYPC* expression and methylation are displayed in Figures 4(d)–4(f) . The expression of *PGR* and *GYPC* is shown in Figure S2. Since the *VTCN1* gene possesses the highest correlation coefficient between methylation and expression, we selected the *VTCN1* gene for the follow-up study.

### 3.4. The Immune Score

In the low immunity group, the stromal score, immune core, and estimate score are −1257.90 ± 283.05, −378.62 ± 401.33, and −1545.68 ± 608.60, respectively. In the high immunity group, stromal score, immune score, and estimate score are −539.79 ± 1241.98, 930.89 ± 583.08, and 462.35 ± 834.37, respectively. The survival curve based on the above immunity score showed that a higher immune score indicated a better prognosis (*P* < 0.05). The stromal score and estimate score showed no statistical significance (*P* > 0.05).

### 3.5. The Mechanism of the *VTCN1* Gene in Immune Regulation

**Figure 5:**
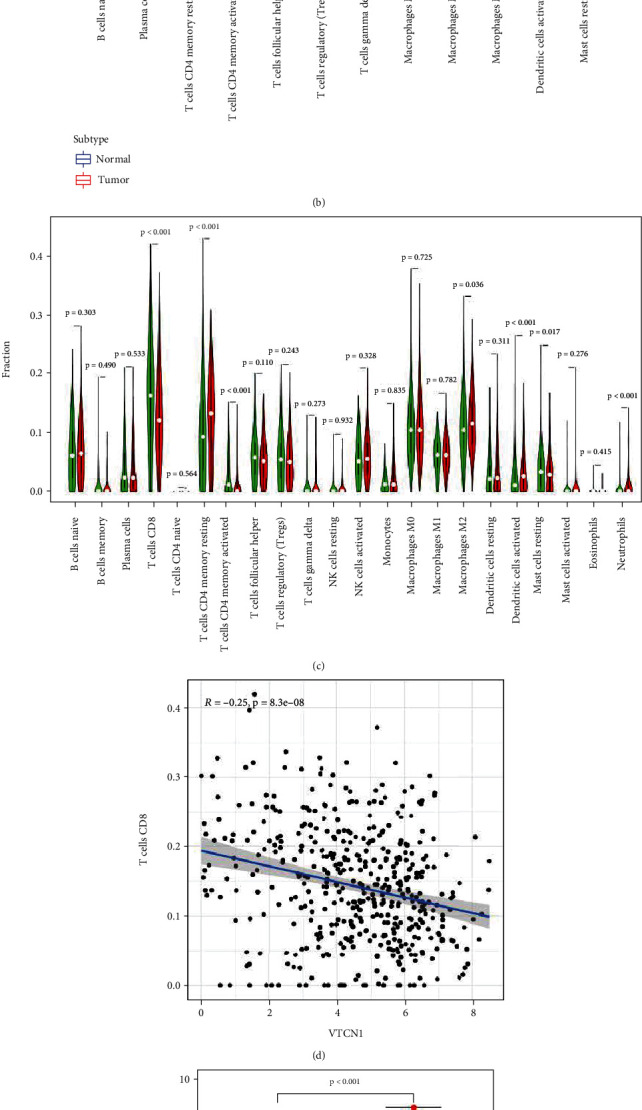
(a) The related proteins of *VTCN1*. (b) The box plot of the immunity cell percentage in the normal and tumor group. (c) The violin plot of the *VTCN1* expression in immunity cell. (d) The correction of T cells CD8 and *VTCN1*. (e)–(g) The expression of *VTCN1* in normal and tumor tissues.

*VTCN1* is closely related to immunity. We further explored the mechanism by which *VTCN1* is involved in endometrial cancer. The relative proteins of *VTCN1* were identified using the STRING database: *B7RP1*, *BTLA*, *CD28*, *CD80*, *CD86*, *CTLA4*, *ICOSL*, *IL4*, *IL6*, and *PDCD1LG2 ***(**Figure 5(a)**)**. These genes are mainly involved in lymphocyte costimulation, regulation of T cell activation, proliferation, B cell activation, and immune response-regulating cell surface receptor signaling pathways (Table 1**)**. The percentage of immune cells in normal and tumor tissues was analyzed. Box plots according to the stromal score, immune score, estimate score, and tumor purity are displayed in Figure 5(b). The box plot of the immune cell percentage in the two groups was analyzed using the “ggpubr” package. The percentages of B native cell and CD4 memory resting T cells and M2 macrophages were clearly lower in the tumor group than in the normal group. Tregs and M1 macrophages were more abundant in the tumor group than in the normal group (*P* < 0.05). Likewise, the higher expression of *VTCN1* exhibited a positive correlation with the abundance of resting memory CD4 T cells, while the higher expression of *VTCN1* was negatively correlated with the abundance of T cells and activated memory CD4 T cells **(**Figure 5(c)**)**. The connection of CD8^+^ T cells and VTCN1 is shown in Figure 5(d). The expression of *VTCN1* was visualized using the “Beeswarm” package. The *VTCN1* expression was higher in the tumor group than in the normal group (Figures 5(e) and 5(f)). The protein expression in the Human Protein Atlas (https://www.proteinatlas.org/) further confirmed the results (Figure 5(g)).

## 4. Discussion

Endometrial cancer is a lethal female reproductive malignant tumor. The incidence of endometrial cancer is usually second only to cervical cancer among gynecological diseases in China [26]. The average age of onset of endometrial cancer is 63 years old, and it usually occurs in postmenopausal women, and in women with obesity and diabetes. Traditional treatments usually include surgical resection, radiotherapy, and chemotherapy. However, it is challenging to treat patients with advanced endometrial cancer, which often has a poor prognosis [27]. Currently, the mechanism of disease and prognostic biomarkers of endometrial cancer is unclear.

DNA methylation and immune cell infiltration often participate in the development of various cancers, including gastric cancer [28], clear cell renal cell carcinoma [29], and colorectal cancer [30]. In our study, we found that high methylation and low expression of *PGR* and *GYPC* were associated with poor prognosis, while low methylation and high expression of *VTCN1* were associated with poor prognosis. In addition, immune regulation driven by the high expression of *VTCN1* in tumors may promote the development of endometrial cancer by inhibiting CD8^+^ T cell infiltration.

*VTCN1* (*B7-H4*) belongs to the B7 family and functions as a cell surface transmembrane protein, negatively regulating the T cell-mediated immune response via interaction with a receptor protein on the surface of T cell to inhibit T cell activation and proliferation and cytotoxic factor production [31, 32]. Several recent studies have shown that *VTCN1* is often overexpressed in tumor tissues of ovarian [33], lung [34], and breast cancers [35]. Miyatake et al. demonstrated that *VTCN1* is overexpressed in high-risk uterine endometrial cancer and negatively correlated with tumor T cell infiltration [36]. In our study, we found that *VTCN1* is downregulated in tumor tissue via DNA methylation analysis. In addition, we performed the CIBERSORT algorithm to analyze the immune cell distribution in normal and tumor tissues. Subsequently, the relationship between *VTCN1* expression and immune cell infiltration was analyzed using bioinformatic methods. The results indicated that the expression of *VTCN1* is inversely correlated with CD8^+^ T cell infiltration.

## 5. Conclusion

In summary, our study first revealed the mechanism of endometrial cancer combining DNA methylation and immune cell infiltration. Hypomethylation of the *VTCN1* promoter leads to its high expression, which can cause tumor development by inhibiting CD8^+^ T cell infiltration. Furthermore, the *VTCN1* expression was higher in the tumor group than in the normal group, and hypomethylation and high expression of *VTCN1* indicated poor prognosis. Our study explains the mechanism of immune infiltration and provides potential targets for the diagnosis and prognosis of endometrial cancer.

## Figures and Tables

**Table 1:** The GO analysis of VCTN1-related genes.

Gene lists	GO analysis
IL4/IL6	Lymphocyte costimulation
B7RP1	Regulation of T cell activation
BTLA	Regulation of T cell proliferation
CD28 CTLA4	Control of immune tolerance by vasoactive intestinal peptide
CD80	Cell adhesion molecules (CAMs)
CD86	B cell activation
ICOSL PDCD1LG2	Immune response-regulating cell surface receptor signaling pathway

## Data Availability

The datasets analyzed during the current study are available in the TCGA and UCSC repository.

## Abbreviations

GO: Gene Ontology
KEGG: Kyoto Encyclopedia of Genes and Genomes
DEGs: Differentially expressed genes.

## Supplementary Materials

Supplementary 1 Figure S1: the volcano plot of differentially expressed genes.

Supplementary 2 Figure S2: the expression of *PGR* and *GYPC* in normal and tumor tissues.
