# Supplementary figures and images for: Integrated Bioinformatic Analysis of DNA Methylation and Immune Infiltration in Endometrial Cancer
Source: Biomed Res Int. 2022 Jun 20;2022:5119411. doi: 10.1155/2022/5119411 (PMC9237709; doi:10.1155/2022/5119411)

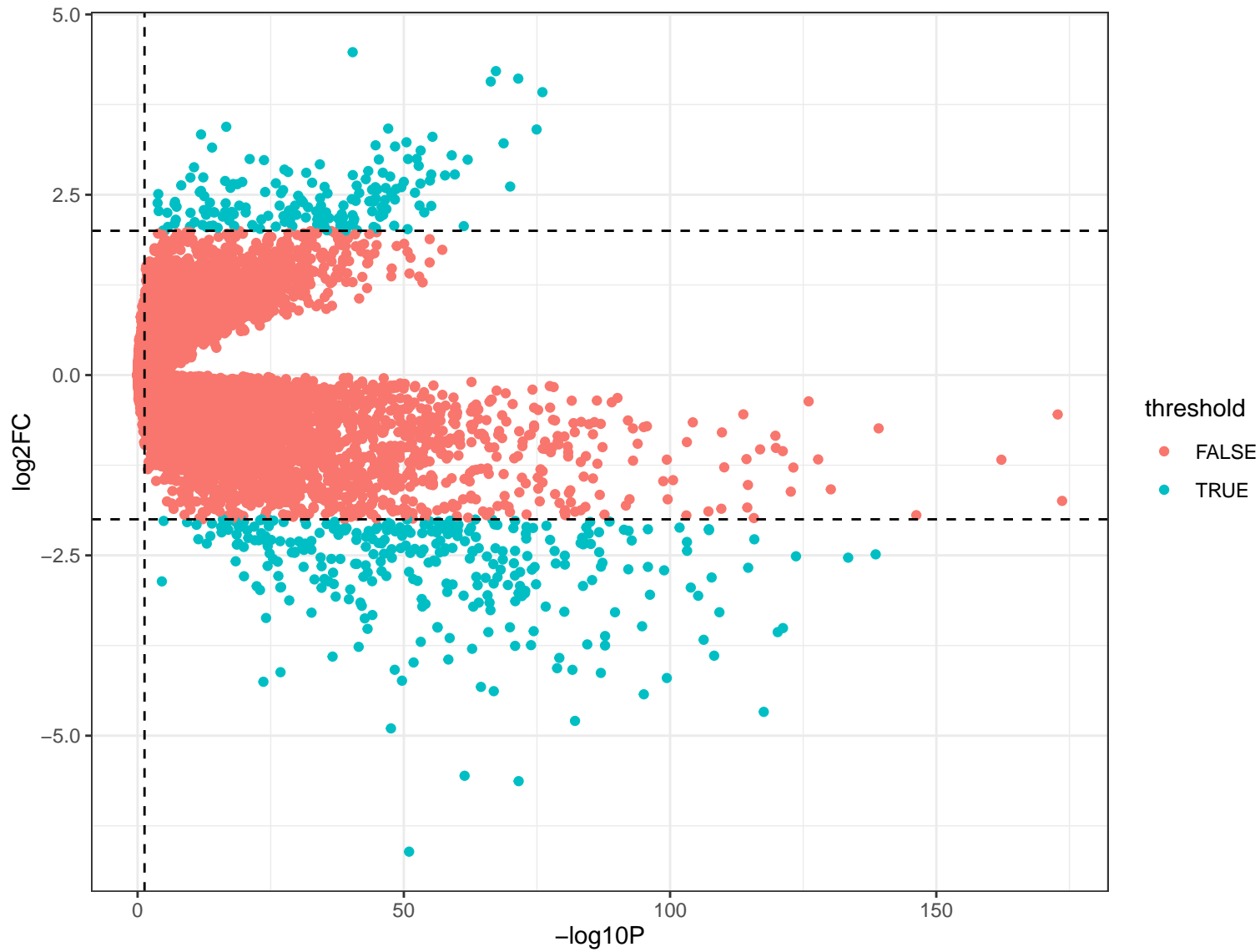

Supplement: Supplementary 1 — Figure S1: the volcano plot of differentially expressed genes. [file 5119411.f1.pdf]

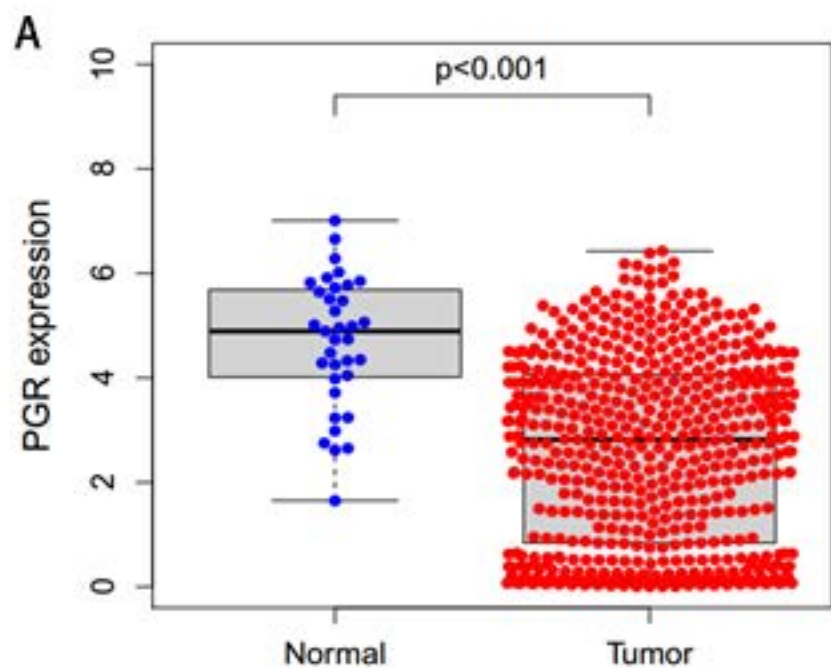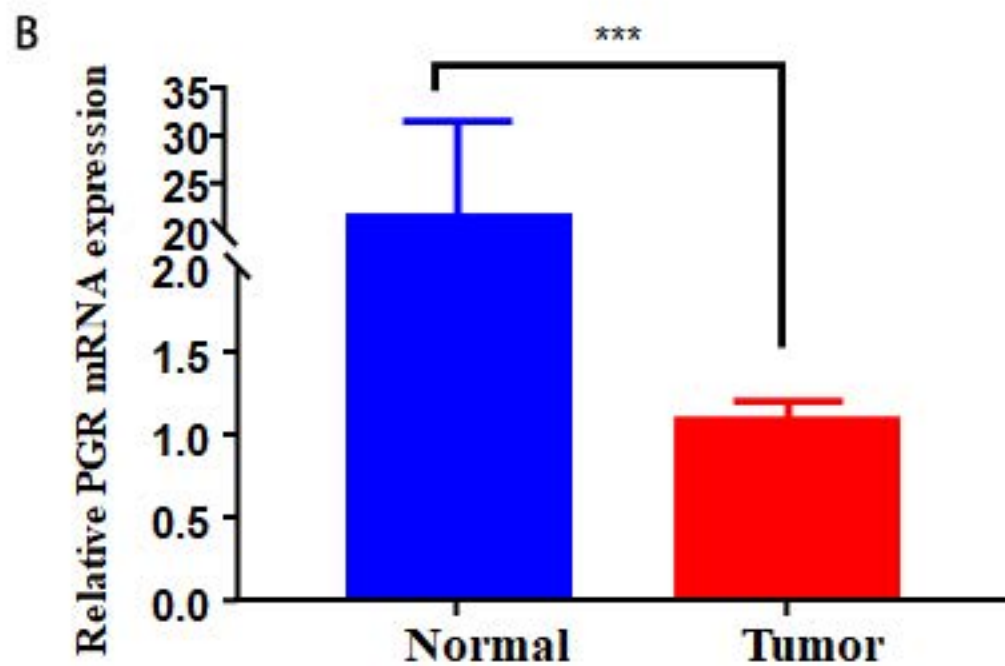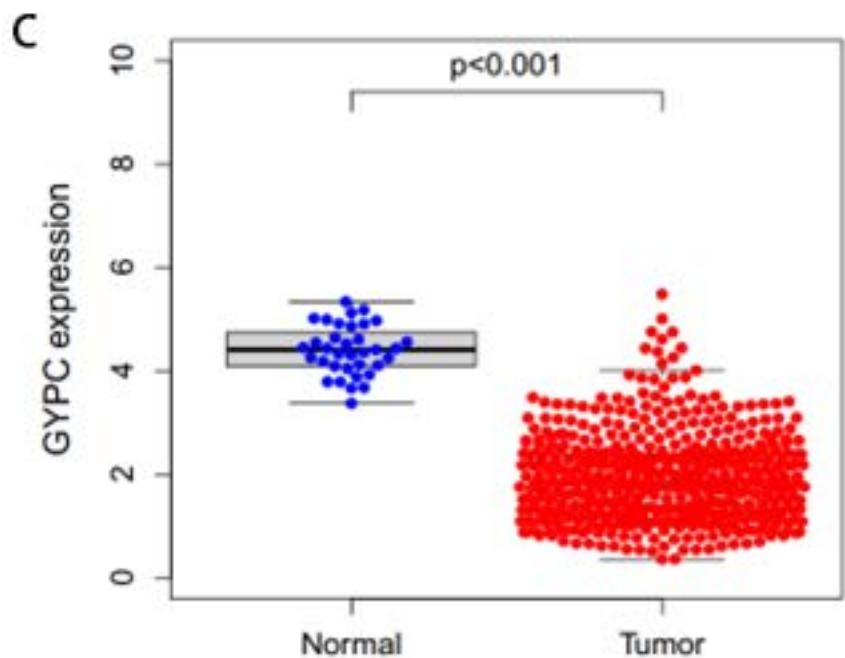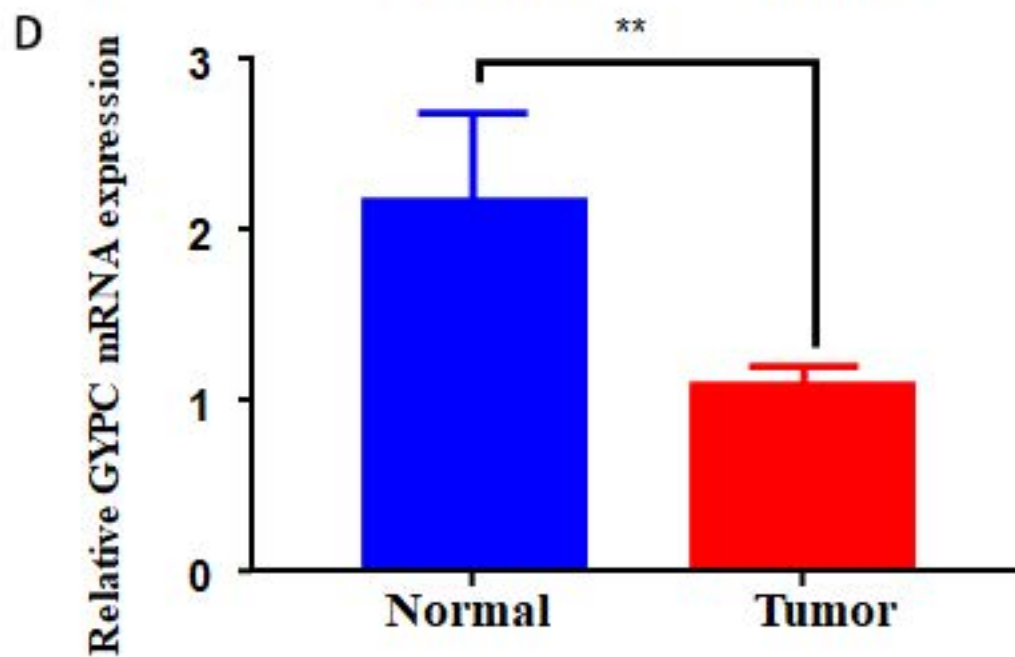

Supplement: Supplementary 2 — Figure S2: the expression of PGR and GYPC in normal and tumor tissues. [file 5119411.f2.pdf]
